# Tortuous Carotid Artery Extended to Neck Level IIb Mimicking the Metastatic Mass

**DOI:** 10.1155/2016/1376926

**Published:** 2016-11-07

**Authors:** Lokman Uzun, Oğuz Kadir Eğilmez, M. Tayyar Kalcioglu, Muhammet Tekin

**Affiliations:** ^1^Department of Otorhinolaryngology, Istanbul Medeniyet University Faculty of Medicine, Istanbul, Turkey; ^2^Malkara State Hospital, Tekirdağ, Turkey

## Abstract

Specifically in neck level IIb, the expected normal anatomy does not contain any vital structures and consequently it might direct a surgeon to perform rapid surgical dissection of tissues. Therefore aberrant anatomy of the vessels in the patients may be overlooked during neck dissection. Unexpected and potentially devastating injuries can be avoided by respecting the possible aberrant anatomy in any level of the neck. In this case report, a 74-year-old man was presented with laryngeal carcinoma who was treated with laryngectomy and bilateral neck dissection. During the left side neck dissection, tortuous internal carotid artery imitating a metastatic mass was unexpectedly encountered in level IIb. As in this case, surgeons should keep in mind possible aberrant anatomy during the neck dissection and perform surgery staying in surgical principles to be safe for an unforeseen and potential dangerous injuries.

## 1. Introduction

Specifically in neck level IIb, the expected normal anatomy does not contain any vital structures and consequently it might direct a surgeon to perform rapid surgical dissection of tissues. Therefore aberrant anatomy of the vessels may be overlooked during neck dissection. Unexpected and potentially devastating injuries can be avoided by respecting the potential unexpected anatomy of the neck [1].

In this presentation, a male patient with tortuous internal carotid artery (ICA) which was mimicking a metastatic mass in neck level IIb was reported and the importance of anatomic variations was emphasized.

## 2. Case Report

**Figure 1:**
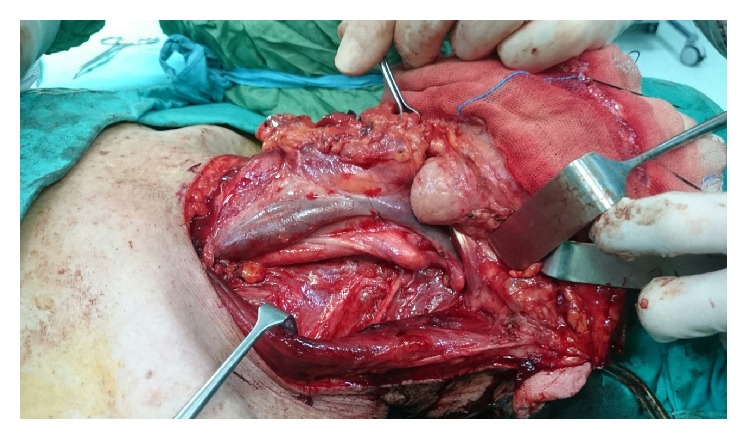
During the neck dissection, tortuous internal carotid artery mimicking a metastatic mass in neck level IIb was detected.

A 74-year-old man was admitted to our clinic with dysphonia and dyspnea. In laryngoscopic examination, an ulcerovegetating mass was seen in laryngeal surface of epiglottis, left ventricular fold, and left vocal fold. The mass exceeds the midline and extends to the right side of the laryngeal surface of epiglottis. On the left side, the movement of arytenoid could not be observed. Histopathologic examination of the biopsy specimen was evaluated as squamous cell carcinoma of the larynx. MR imaging showed a mass with contrast enhancement in the larynx. Total laryngectomy and functional neck dissection for both sides of the neck were planned. During the neck dissection on the left side, though there was a lymphadenopathy in level IIa, it was decided to add the level IIb to the dissection. During the dissection of level IIb, we detected a mass embedded into lymphoadipose tissue by palpation. Though the mass was pulsatile, careful dissection was performed and it was understood that the pulsatile mass was a continuation of the tortuous internal carotid artery mimicking a metastatic mass at the first glance (Figure 1). The angulation of the vessel was less than 30 degrees so the tortuosity of the internal carotid artery was graded as grade 3 according to Metz et al.'s classification [2]. Because of this anatomical variation, the rest of the operation was performed meticulously to avoid any damage to the carotid artery and surrounding tissue. Operation and postoperative period were uneventful and passed without any complications.

## 3. Discussion

Aberrant routes of the extracranial ICA are widely known and not unusual anatomic variations, but their clinical effect is underestimated. In the literature, it is possible to read previously reported several anatomic variations of ICAs. Prominent parapharyngeal ICA anomalies can be found in up to 5% of the general population and may place the artery into close correlation with the pharyngeal wall, where it is at risk of damage particularly during surgical interventions [1, 3]. Tortuous ICA, ICA located in the tympanic cavity, a retropharyngeal course of an ICA, and carotid bifurcation's absence that resulted an ICA agenesis are described ICA anomalies [4–7].

There are two provoking factors that appear to play a role in the development of ICA aberrations: age-related degenerative changes in the vessel wall and embryological maldevelopment, as well as the combination of both [3]. Because, in young children, pronounced kinking and coiling can also be found often bilaterally with the same age-independent incidence, a congenital malformation seems likely. During embryological development, a loop is formed at the junction of the ICAs and the third paired dorsal aortae which is the origin of these vessels. While the heart is descending into the mediastinum, this loop usually uncoils, but aberrations in this step may inhibit straightening of the artery and end in an anomalic ICA that mostly shows an anteromedial tortuosity at the level of the oropharynx [3]. Extracranial ICA anomalies are clinically silent in the most of cases. When recognized, majority of them are diagnosed incidentally by radiological investigations and infrequently during physical examinations or surgery. In clinically negative neck, the preservation of level IIb in selective neck dissection for laryngeal squamous cell carcinoma is recommended [8]. If positive lymph nodes are found in level IIa, level IIb dissection is performed.

Anomalies that are located anatomically in an important region and have a major risk for complications should be recognized by radiologists in the preoperative imaging taken before the surgery and the surgeon should be warned for such anomalies. In fact, the surgeon should review the imaging to guarantee him/herself despite the report of the radiologist. MRI was taken for our patient before the surgery but neither the radiologist nor we paid attention for this anomaly in the images. Briefly, preoperative imaging is an important tool for surgeons while planning the surgery.

ICA anomalies are noteworthy because when ICA is tortuous, the vessel lacks a definitive muscular layer and will be softer than normal [9]. Also, reduced axial tension or elongation and weakening of the arterial wall are associated with tortuous arteries [10]. So surgical intervention on carotid artery with weakened wall carries more life-threatening risks. As in our case, the possibility of aberrant anatomy during the neck dissection should be kept in mind and surgery should be performed staying in surgical principles to be safe for an unforeseen and potential dangerous injuries.
